# Oral Hydrogels that Balance Microbiome for Tumor Treatment

**DOI:** 10.34133/research.0494

**Published:** 2024-10-02

**Authors:** Chu Jiang, Huajie Liu, Yinan Zhang

**Affiliations:** School of Chemical Science and Engineering, Tongji University, Shanghai, China.

## Abstract

Intervening in the microbial environment holds promise for enhancing antitumor efficacy by reshaping the tumor microenvironment, yet few strategies have been reported. In a study led by Zou and coworkers, oral hydrogels are introduced to regulate the microbiota balance in the intestines and tumors, triggering an antitumor immune response. This work presents a microbiota-targeted drug delivery system that demonstrates notable efficacy in colon targeting and colon retention for the treatment of colorectal cancer. This represents a significant clinical advancement in treating colorectal cancer, which is particularly vulnerable to microbial infiltration.

The commensal of bacteria and other microorganisms, collectively known as the microbiota, that colonize the epithelial surfaces of our body has been shown to produce small molecules and metabolites with both local and systemic effects on cancer onset, progression, and therapy response. Within these microbiotas, the symbiotic balance of the gut microbiome is particularly crucial for overall biological health [1]. Clinical data have indicated that various intestinal bacteria can enter the blood circulation through the mucosal system and accumulate in different types of tumor tissues, thus creating an “intratumoral microbiome” as the tumor advances [2,3]. Notably, the intestinal and tumor microbiota significantly influence the occurrence, development, and therapeutic response of tumors by modulating carcinogenic signaling pathways, promoting mutations, modifying the metabolism of chemotherapy drugs, and impacting the host immune response [4].

Most studies on the microbiome have relied on traditional preclinical mouse models to establish connections between microbial species and cancer phenotypes, revealing a profound influence of the microbiota on the efficacy of cancer treatments [5]. Given the intricate relationship between the microbiome and cancer, it is crucial to prioritize the development of antitumor treatments that target the tumor-associated microbiome. This is particularly pertinent for solid tumors such as colorectal cancer, which are susceptible to microbial invasion [6].

In addressing these internal relations, a recent study conducted by Zou et al. at Southern Medical University has presented findings on an oral hydrogel possessing colon-targeting and retention properties. Compared with layered double hydroxide nanoparticle-reinforced alginate and hyaluronic acid hydrogel beads [7] and a hydrogel microsphere with colon-targeting and adhesive properties [8], which have been studied recently, this hydrogel has been shown to modulate the balance between beneficial and harmful bacteria in the intestinal tract and tumors, and activate an antitumor immune response [9]. To investigate the antitumor effect of this oral hydrogel, they utilized subcutaneous and in situ colorectal cancer mouse models, which both showed favorable outcomes. The oral hydrogel can maintain a stable hydrogel structure and excellent antitumor efficacy at 4 °C for 7 d. This study introduces a promising treatment modality for colorectal cancer, a condition particularly vulnerable to microbial infiltration.

In their study, the authors developed an oral inulin-based hydrogel with hollow manganese dioxide nanocarriers and the chemotherapy drug oxaliplatin loaded (Oxa@HMI), demonstrating colon-targeting and colon retention properties (Fig. 1). The selection of inulin as the gel matrix was based on its dual advantages. First, the inulin matrix promotes the accumulation and concentration of the active ingredient at the lesion site through enhanced bioadhesion and prolonged colon retention time. Second, it facilitates the targeted exposure of internal cargos at the site of colorectal cancer, which are degraded into Mn^2+^ and O_2_ in the acidic tumor microenvironment and release supported oxaliplatin. During the process, the specific degradation of the inulin gel matrix by inulinase, secreted by beneficial bacteria in the colon, leads to the production of short-chain fatty acids. Subsequently, these fatty acids initiate a sequence of antitumor immune responses by regulating the balance between harmful and beneficial bacteria. The authors astutely utilized the concentration accumulation of the inulin matrix to instigate the organism’s anticancer response and the synergistic effect of chemical drugs, thus realizing tumor growth inhibition.

**Fig. 1.**
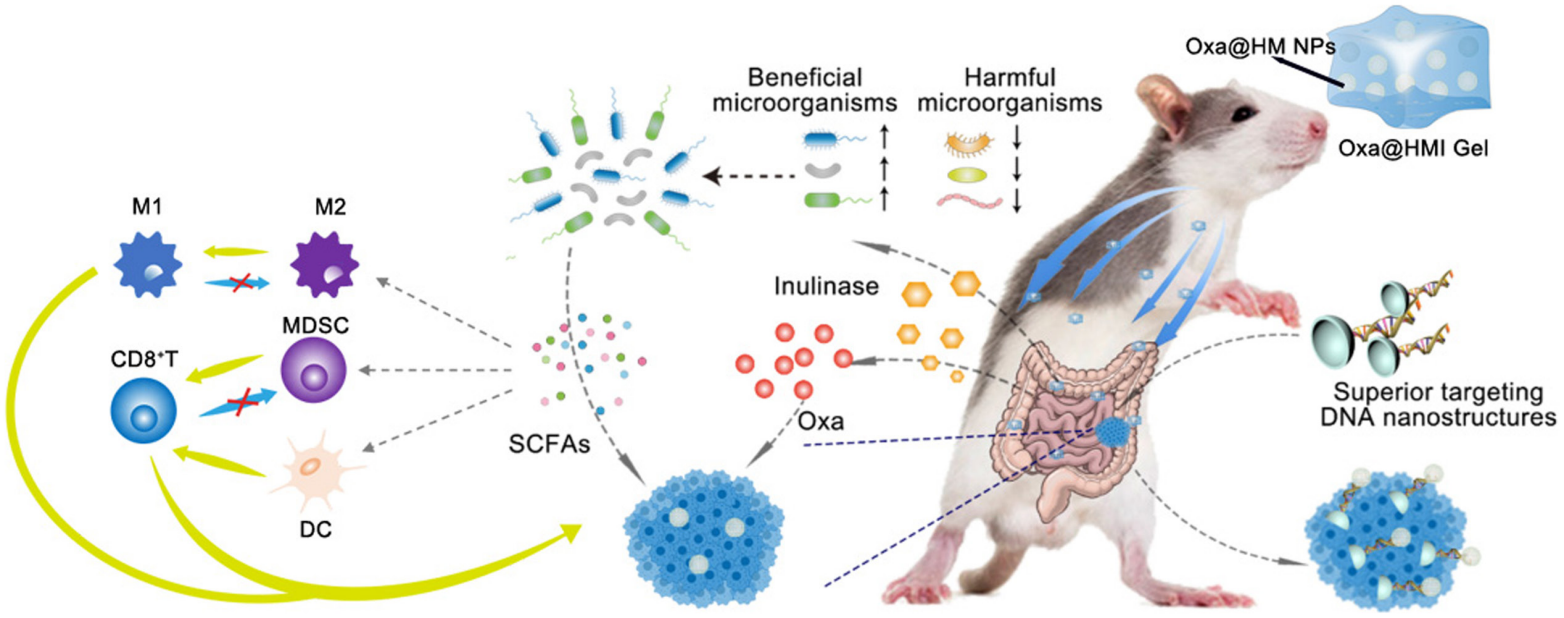
Colon tumor suppression mechanism of Oxa@HMI Gel via regulating microbiota and immune microenvironment.

To elucidate the impact of orally administrated hydrogel on the antitumor effect and the applicability of oral hydrogel in the treatment of multiple colorectal tumor models, the authors conducted an investigation using orthotopic colorectal tumor-bearing mice and subcutaneous colorectal tumor-bearing mice to assess the efficacy of the treatment. Their findings suggest that the orally administrated hydrogel exhibits specific colon-targeting behavior, attributed to the selective degradation of the inulin protective layer by inulinase solely within the colorectal region. The adhesive properties of the hydrogel facilitate its adhesion to the intestinal mucosal layer, thereby extending the residence time of nanomaterials within the colon and promoting increased accumulation and concentration of the nanomaterials within colon tumors. Consequently, by modulating the microbial environment of the tumor tissue, the orally administered hydrogel enhances the efficacy of chemotherapy and induces an antitumor immune response, thereby demonstrating notable antitumor efficacy in both murine models.

To assess the alterations in microbiota within the gut and tumor tissue of mice with in situ colorectal cancer, the authors utilized high-throughput 16S ribosomal RNA (rRNA) gene sequencing to characterize the relative abundance of beneficial bacteria and harmful bacteria. The results indicated that oral administration of hydrogel significantly augmented the relative abundance of *Lactobacillus*, *Lachnospiraceae*, *Roseburia*, and *Akkermansia* while markedly decreasing the relative abundance of *Alistipes*, *Helicobacter*, *Clostridia*, and *Escherichia-Shigella*, subsequently achieving microbiota normalization and activating antitumor immune responses. Furthermore, the authors analyzed the expression levels of relevant receptors and immune cells in colorectal tumor-bearing mice. The results revealed that the microbial metabolite, short-chain fatty acid, triggered a sequence of antitumor immune responses by binding to G protein-coupled receptor 43 (GPR43) in the gut, including significantly increasing the levels of infiltrating CD8^+^, IFN-γ^+^ CD8^+^ and CD4^+^ T cells within tumor tissues, instigating dendritic cell maturation, facilitating the polarization of tumor-associated M2 macrophages into pro-inflammatory M1 macrophages, and diminishing the occurrence of immunosuppressive myeloid-derived suppressor cells (MDSCs) [10]. Additionally, the butyrate produced stimulates the expression of ID2 in CD8^+^ T cells through inhibition of histone deacetylases (HDACs). The up-regulated ID2 induces the expression of interleukin-12 (IL-12) receptors on the surface of CD8^+^ T cells, thereby modulating IL-12 signaling, promoting the infiltration of CD8^+^ T cells into tumor tissues, enhancing IFN-γ secretion, and ultimately strengthening T cell-mediated antitumor immune responses. Notably, the Oxa@HMI hydrogel demonstrated favorable biosafety. Throughout the treatment, the body weight of mice in both the orthotopic and subcutaneous tumor groups elevated consistently, and no noteworthy harm was observed in the hematoxylin and eosin (H&E) staining of major organs within each group.

The study highlights the efficacy of an oral inulin-based hydrogel featuring colon-targeting and retention properties, which effectively modulates the microbial balance in the intestines and tumors. The observed antitumor effect suggests potential applicability in the treatment of microbially vulnerable colorectal cancer. Based on this, utilizing HMI hydrogel for the targeted delivery of olsalazine, sulfasalazine, and sinomenine to the colon shows potential applications in anti-inflammatory treatment for inflammatory bowel disease and the repair of intestinal damage caused by intestinal sepsis. It also has potential therapeutic effects on breast cancer, gastric cancer, and cervical cancer, which are closely related to the imbalance of gut and tumor-associated microbiota. Furthermore, the prospect of integrating DNA nanostructures into this system to enhance targeting warrants investigation as a promising next step for researchers. Particularly, DNA origami-based nanodevices that are equipped with both targeting and medication components have displayed prominent potential in personalized precision medicine, with markedly improved therapeutic efficacy [11]. By integrating specific responsive DNA components within hydrogels, the prospect of developing sophisticated biomedical hydrogels capable of adapting to varying environmental conditions and assuming corresponding configurations for programming drug release profiles becomes a tangible reality. Besides, DNA hydrogels synthesized by rolling circle amplification (RCA) are drawing paramount attention from nanomedicine for their tunable properties and reasonable cost [12].

## References

[1] Brown JM, Wilson WR. Exploiting tumour hypoxia in cancer treatment. Nat Rev Cancer. 2004;4(6):437–447. 15170446 10.1038/nrc1367

[2] Elinav E, Garrett WS, Trinchieri G, Wargo J. The cancer microbiome. Nat Rev Cancer. 2019;19(7):371–376. 31186547 10.1038/s41568-019-0155-3 PMC6700740

[3] Jiang X, Zhang Y, Wang H, Wang Z, Hu S, Cao C, Xiao H. In-depth metaproteomics analysis of oral microbiome for lung cancer. Research. 2022;2022:9781578. 36320634 10.34133/2022/9781578 PMC9590273

[4] Coker OO, Nakatsu G, Dai RZ, Wu WKK, Wong SH, Ng SC, Chan FKL, Sung JJY, Yu J. Enteric fungal microbiota dysbiosis and ecological alterations in colorectal cancer. Gut. 2019;68(4):654–662. 30472682 10.1136/gutjnl-2018-317178 PMC6580778

[5] McQuade JL, Daniel CR, Helmink BA, Wargo JA. Modulating the microbiome to improve therapeutic response in cancer. Lancet Oncol. 2019;20(2):e77–e91. 30712808 10.1016/S1470-2045(18)30952-5 PMC12908161

[6] Iida N, Dzutsev A, Stewart CA, Smith L, Bouladoux N, Weingarten RA, Molina DA, Salcedo R, Back T, Cramer S, et al. Commensal bacteria control cancer response to therapy by modulating the tumor microenvironment. Science. 2013;342(6161):967–970. 24264989 10.1126/science.1240527 PMC6709532

[7] Nguyen NT, Nguyen BT, Ho TN, Tran CD, Tran TH, Nguyen HH, Nguyen HP, Huynh NT, Li Y, Phan VHG, et al. Orally ingestible medication utilizing layered double hydroxide nanoparticles strengthened alginate and hyaluronic acid-based hydrogel bead for bowel disease management. Int J Biol Macromol. 2024;269(Pt 1): Article 132122. 38718992 10.1016/j.ijbiomac.2024.132122

[8] Yang X, Nie W, Wang C, Fang Z, Shang L. Microfluidic-based multifunctional microspheres for enhanced oral co-delivery of probiotics and postbiotics. Biomaterials. 2024;308: Article 122564. 38581763 10.1016/j.biomaterials.2024.122564

[9] Li L, He S, Liao B, Wang M, Lin H, Hu B, Lan X, Shu Z, Zhang C, Yu M, et al. Orally administrated hydrogel harnessing intratumoral microbiome and microbiota-related immune responses for potentiated colorectal cancer treatment. Research. 2024;7:0364. 38721274 10.34133/research.0364 PMC11077293

[10] Han K, Nam J, Xu J, Sun X, Huang X, Animasahun O, Achreja A, Jeon JH, Pursley B, Kamada N, et al. Generation of systemic antitumour immunity via the in situ modulation of the gut microbiome by an orally administered inulin gel. Nat Biomed Eng. 2021;5(11):1377–1388. 34168321 10.1038/s41551-021-00749-2 PMC8595497

[11] Yin J, Wang S, Wang J, Zhang Y, Fan C, Chao J, Gao Y, Wang L. An intelligent DNA nanodevice for precision thrombolysis. Nat Mater. 2024;23(6):854–862. 38448659 10.1038/s41563-024-01826-y

[12] Yao C, Zhang R, Tang J, Yang D. Rolling circle amplification (RCA)-based DNA hydrogel. Nat Protoc. 2021;16(12):5460–5483. 34716450 10.1038/s41596-021-00621-2

